# A comparison of femoral component rotation after total knee arthroplasty in Kanekasu radiographs, axial CT slices and 3D reconstructed images

**DOI:** 10.1007/s00256-020-03702-7

**Published:** 2021-01-04

**Authors:** Emma L. Robertson, Martin Hengherr, Felix Amsler, Michael T. Hirschmann, Dominic T. Mathis

**Affiliations:** 1 Department of Orthopaedic Surgery and Traumatology, Kantonsspital Baselland (Bruderholz, Liestal, Laufen), 4101 Bruderholz, Switzerland; 2 University of Basel, 4051 Basel, Switzerland; 3 Department of Radiology, Kantonsspital Baselland (Bruderholz, Liestal, Laufen), 4101 Bruderholz, Switzerland; 4 Amsler Consulting, 4059 Basel, Switzerland

**Keywords:** Total knee arthroplasty, Rotational alignment, 2D computer tomography, Kanekasu, Reliability, Prediction

## Abstract

**Objective:**

To compare the posterior condylar angle measured with Kanekasu radiograph and 2D-CT with the gold standard 3D-CT following primary total knee arthroplasty (TKA).

**Methods:**

Eighty-two knees with pain following TKA were included in this retrospective study. Two independent raters measured the anatomical and surgical posterior condylar angles twice on each Kanekasu radiograph and 2D-CT. These measurements were compared against the 3D-CT measurement. The intra- and interrater reliability of the Kanekasu radiograph and 2D-CT and the correlation with 3D-CT were calculated.

**Results:**

The intra- and interrater reliability for measurements of the anatomical posterior condyle angle for the Kanekasu radiograph and the 2D-CT were excellent for both raters (0.85–0.92). For the less experienced rater 1, the intrarater reliability was significantly better for 2D-CT than Kanekasu radiograph for measuring both the surgical (*p* < 0.01) and anatomical posterior condyle angles (*p* < 0.05). For the experienced rater 2, the intrarater reliability was significantly better for Kanekasu radiograph than 2D-CT for measurement of the surgical posterior condyle angle (*p* < 0.05). The correlation with 3D-CT is higher in 2D-CT than in Kanekasu radiograph (*p* < 0.01). While the Kanekasu radiograph predicts the 3D-CT angle with 65.9%, 2D-CT can measure the true angle with 82.9% certainty.

**Conclusion:**

Measurements using the anatomical transepicondylar axis are easier to replicate compared to the surgical transepicondylar axis. In comparison with the gold standard 3D-CT, 2D-CT showed a significantly higher correlation with 3D-CT than the Kanekasu measurements. If 3D-CT is available, it should be preferred over 2D-CT and Kanekasu view radiograph for femoral component rotation measurements.

## Introduction

Femoral component position in total knee arthroplasty (TKA) is considered to be an important factor for clinical outcome [1–3]. The position of the femoral component significantly affects the acting forces on the knee joint and associations have been found between femoral component malrotation and a variety of complications including patellofemoral maltracking, anterior knee pain, flexion instability, and wear or loosening caused by abnormal torsional stress on the tibial component [4–9].

Femoral component rotation is defined by the posterior condylar axis (PCA) relative to the surgical [10, 11], or anatomical transepicondylar axis (sTEA or aTEA) [12]. In literature, this angle is referred to as the posterior condylar angle [10, 11, 13]. A number of different methods of assessing femoral component rotational alignment have been described and a variety are used in clinical practice [13, 14]. Moreover, there is controversy amongst the orthopaedic community whether sTEA or aTEA should be used as a reference. Both axes are distinct concepts: While sTEA is regarded as a more accurate reference to determine intraoperative rotation of the femoral component [6], aTEA is less difficult to identify on CT slices [15, 16]. Standard radiographs of the knee may demonstrate more extreme incidences of femoral component malpositioning; however, inaccurate measurements can occur due to non-standardised positioning of the leg or issues with magnification [17, 18]. Computer tomography (CT) planar images (hereafter referred to as 2D-CT) and CT 3D volumetric, surface rendered images (hereafter referred to as 3D-CT) are also applied in clinical practice using various landmarks (i.e., sTEA, aTEA, PCA) for assessing femoral component rotation [13].

A recently published systematic review comparing intraoperative techniques for determination of femoral component placements with postoperative component positioning imaging emphasized the superiority of 3D-CT compared to 2D-CT and plain radiographs in terms of reproducibility, accuracy and reliability [14]. Thus, 3D-CT has become the gold standard in modern medicine [13, 17]. However, CT scans are associated with an increased radiation exposure for the patient in addition to significant financial cost.

In 2005, Kanekasu et al. [19] described a method for assessing rotational alignment following TKA using axial radiography of the distal femur. The angle between two tangents, the aTEA and the PCA, is measured [19]. The original study by Kanekasu et al. [19] reported that this imaging method was found to be comparable to 2D-CT for the assessment of femoral component rotation.

Berger et al. [5] developed a method for assessing femoral rotational alignment by measuring the angle between the tangent of the sTEA and the PCA on transverse 2D-CT images postoperatively. The authors postulated that 2D-CT can accurately confirm the diagnosis of rotational malalignment in patients with a malfunctioning TKA and patellofemoral pain, as well as aiding planning of revision surgery.

However, to date, the reliability of Kanekasu view radiographs in assessing femoral component rotational alignment compared to both 2D- and 3D-CT has not yet been established. Therefore, the primary aim of the present work was to establish the intra- and interrater reliability and absolute differences of posterior condylar angle measurements between Kanekasu view radiographs and 2D-CTs by comparing it with the gold standard 3D-CT following primary TKA. Secondary aim was to compare the findings obtained depending on the use of the aTEA or sTEA.

It was our primary hypothesis that 2D-CT shows stronger correlation with the gold standard than Kanekasu view radiograph in measuring the posterior condyle angle. A secondary hypothesis for this study was that 2D-CT has a higher intra- and interrater reliability than Kanekasu view radiograph.

## Materials and methods

A consecutive number of 82 knees from 78 patients (male:female 27:51, mean age at the time of Kanekasu view radiograph 64 ± 10 years, range 24–83 years, left:right 43:39), who underwent primary TKA from 2004 to 2019 were included in this single-centre, retrospective cohort study. Data was prospectively collected from a specialised knee centre (tertiary hospital) in which the patients presented between 2014 and 2020 with persistent pain or instability following primary TKA. As a specialised knee centre with focus on painful TKAs, most of the patients were referred from other surgeons to our clinic. Therefore, a total of 55 different surgeons have performed the primary TKAs.

All included patients routinely underwent both imaging modalities (Kanekasu view radiograph and axial 2D-CT) during assessment for pain or instability. Another prerequisite for inclusion in the study was a completed 3D-CT examination. Patients with a history of trauma to the affected knee or who had undergone revision surgery were excluded from this study (*N* = 12).

The study was approved by the local ethics committee (2020-01382) and was performed in accordance with the ethical standards of the responsible committee and with the guidelines of the Helsinki Declaration of 1975, as revised in 2008. A written institutional general consent was signed by every patient.

Two independent raters measured femoral TKA rotation based on posterior condylar angles on Kanekasu view radiographs and 2D axial CT slices twice with a 4-week interval between interpretations in a random order. An interval of 2 weeks was also kept between the interpretations of the two imaging modalities. The two raters were blinded to both their own previous measurements and the measurements of the other rater. The intra- and interrater reliability was then calculated. Rater one (R1) was an orthopaedic research fellow (E.R.) and rater two (R2) was a fully trained and nuclear-medicine and musculoskeletal specialised radiologist (M.H.) with an image interpretation experience of 3 and 25 years, respectively. For 3D-CT, measurements had been previously recorded by one fully trained, nuclear-medicine and musculoskeletal specialized radiologist with an experience of 25 years at the specialized knee centre and were considered as the gold standard measurement, due to previously reported excellent intra- and interrater reliability [17].

The measurements in Kanekasu view radiographs were performed according to the protocol described by Kanekasu et al. [19] in 2005 using the aTEA for the assessment of the anatomical posterior condyle angle (“twist angle”). These measurements were complemented by also determining the sTEA and thus the surgical posterior condyle angle. The technical settings for the Kanekasu view radiograph were as follows: The patients sat on a table (72 cm high) with their lower legs hanging down at neutral rotation. The position of neutral rotation was taken as the position at which the subject naturally placed his or her knees and lower legs without any specific instruction. The positions of the knee were adjusted so that the central ray of the x-ray beam (Digital Diagnost VM, 650 mA, 150 kV, Philips Medical Systems, Zürich, Switzerland) was directed to the centre of the patella. The x-ray beam was directed at a 15° upward angle. The distance between the x-ray tube and the image receptor was set at 100 cm. The default setting was at 60 kV [19].

Correspondingly, all measurements performed in 2D-CT scans were done based on the protocol described by Berger et al. [5] using the sTEA and then complemented by the aTEA. All patients were scanned on a dual-source CT scanner (Symbia T16, Erlangen, Germany), which consists of a pair of low-energy, high-resolution, parallel hole collimators and an integrated 16-slice CT scanner (collimation of 16 × 0.75 mm). A standardised imaging protocol was used which was based on the Imperial Knee Protocol [20]. CT slice thickness was 0.75 mm; energy window was centered at 140 kV.

**Fig. 1:**
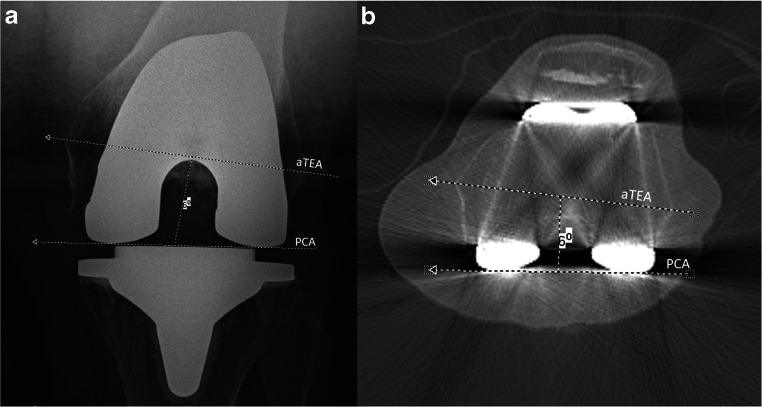
The anatomical posterior condylar angle formed by the anatomical transepicondylar axis (aTEA) and the posterior condylar axis (PCA) on Kanekasu view radiograph (**a**) and 2D axial computer tomography (**b**) of a 59-year-old woman after total knee arthroplasty

**Fig. 2:**
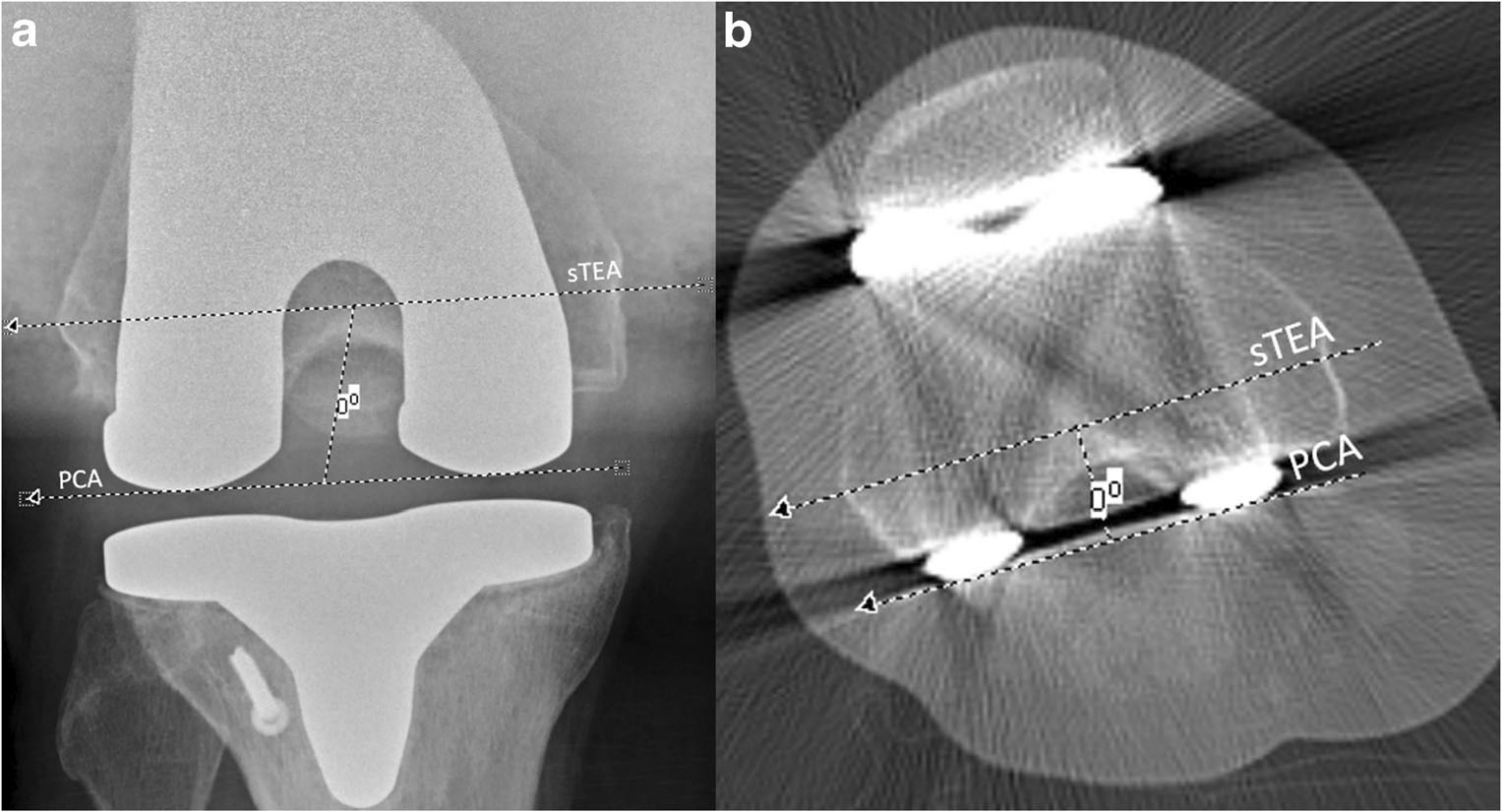
Surgical posterior condylar angle formed by the surgical transepicondylar axis (sTEA) and the posterior condylar axis (PCA) on Kanekasu view radiograph (**a**) and 2D axial computerised tomography (**b**) of a 64-year-old man after total knee arthroplasty

The rotation of the femoral component was established at the level of the femoral epicondyles (in 2D-CT from a single axial slice of the femur in which the femoral epicondyles were most pronounced and visible). The sTEA was plotted as a line drawn from the sulcus of the medial epicondyle to the prominence of the lateral epicondyle. The aTEA was a line between the tips of the medial and lateral epicondyles. The PCA of the femoral component was plotted as a line connecting the posterior margins of the medial and lateral posterior component condylar surfaces. The angle between these two lines was then measured, the anatomical posterior condyle angle between the aTEA and PCA (Fig. 1), and the surgical posterior condylar angle between the sTEA and PCA (Fig. 2) [15, 21]. Hence, both axes, aTEA and sTEA, were separately analysed and compared.

Measurements for Kanekasu view radiographs and 2D-CT were performed on PACS (patient archiving and communication system). PACS automatically calculated the anatomical and surgical posterior condylar angles from the inputted lines and the angle and direction of rotation (internal *+* or external *−*) was documented by the examiner in an encrypted excel document specific to them.

**Fig. 3:**
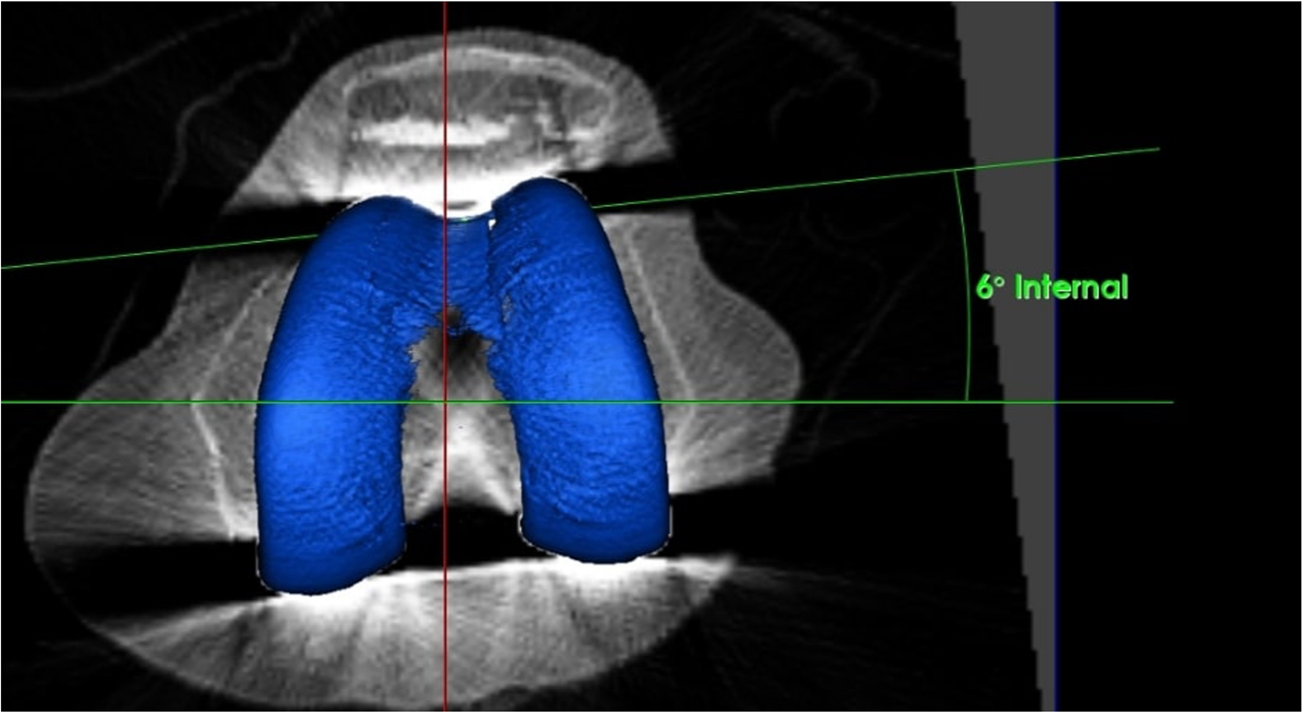
Measurement of femoral component rotation of a 62-year-old man on 3D computer tomography (Orthoexpert®, London, UK) using the anatomical transepicondylar axis (aTEA) and the anterior shield (parallel to the PCA). The measurement shows an internal rotation of 6 degrees in relation to the aTEA

The data from the 3D-CTs was previously assessed on Orthoexpert® (London, UK) by an experienced, fully trained radiologist and the angles were saved on the PACS software (Fig. 3). These angles were extracted for the study and recorded in an encrypted excel spreadsheet. Orthoexpert® uses the anterior part (shield) of the femoral TKA component as reference for the PCA as the anterior femoral shield is parallel to the PCA (Fig. 3). Hence, the posterior condylar angle is defined as the angle between the aTEA and the anterior femoral TKA shield.

All statistical analyses were performed by an independent professional statistician using SPSS statistics for windows, version 26.0 (Armonk, NY: IBM Corp, USA). Sample size was estimated according to the reported estimates for reliability studies using intraclass correlation coefficients (ICCs) [22]. With a given sample size of 80, an effect size of *d* = 0.32 can be detected with a power (1- β) of 80%.

The intrarater reliability was calculated using ICCs and was calculated separately for each rater. The mean value of each rater’s measurements for Kanekasu radiographs and 2D-CT images was calculated, and the interrater reliability was reported. Additionally, systematic differences in the values measured with the three different imaging methods was established using *t* tests. For the purpose of this study, it is assumed that the values from the 3D-CT measurements are correct, and the values of 2D-CT and Kanekasu view radiographs were compared with this. Differences were always interpreted as false for 2D-CT or Kanekasu view radiographs. ICCs were classified according to the system by Rosner, with an ICC higher than 0.75 considered as ‘excellent’, between 0.40 and 0.75 as ‘fair to good’ and below 0.40 as ‘poor’ [23].

## Results

Mean values and findings for intra- and interrater reliability for Kanekasu view radiographs and 2D-CT measurements, anatomical and surgical posterior condyle angle measurements and correlation with 3D-CT, expressed as ICCs are outlined in Table 1. The intra- and interrater reliability for measurements of the anatomical posterior condyle angle for both the Kanekasu view radiograph, and the 2D-CT were excellent for both raters (0.85–0.92).

**Table 1:** Mean values (± standard deviation, SD) for measurements and intra- and interobserver reliabilities expressed as ICCs (intraclass correlation coefficient). Statistically significant p-values are shown in italics. *p* < 0.05 are classed as statistically significant and ≥ 0.05 as not significant. R1, 2; rater 1 and 2; M1, 2; measurement 1 and 2. CI; confidence interval

Comparison	Variable 1		Mean ± SD	Variable 2		Mean ± SD	Mean diff. ± SD	*p*	ICC	95% CI
Intrarater R1	Kanekasu	Surg. M1	− 1.6; 2.78	Kanekasu	Surg. M2	− 1.9; 2.93	0.31; 2.13	0.199	0.721	0.599 – 0.811
		Anat. M1	2.65; 2.84		Anat. M2	2.96; 3.01	− 0.32; 1.6	0.077	0.850	0.777–0.901
	2D-CT	Surg. M1	− 0.45; 2.56	2D-CT	Surg. M2	− 0.46; 2.77	0.01; 1.34	0.934	0.874	0.811–0.917
		Anat. M1	2.96; 2.9		Anat. M2	3.15; 2.79	− 0.18; 1.14	0.148	0.920	0.879- 0 .948
Intrarater R2	Kanekasu	Surg. M1	− 1.72; 2.57	Kanekasu	Surg. M2	− 1.89; 2.33	0.17; 0.97	0.113	0.923	0.882–0.949
		Anat. M1	3.29; 2.52		Anat. M2	3.02; 2.49	0.27; 1.1	*0.030*	0.904	0.854–0.937
	2D-CT	Surg. M1	− 1.22; 2.39	2D-CT	Surg. M2	− 1.43; 2.46	0.21; 1.3	0.153	0.856	0.785–0.904
		Anat. M1	3.07; 2.5		Anat. M2	2.9; 2.63	0.17; 1.39	0.268	0.854	0.783–0.903
Interrater	Kanekasu	Surg. R1	− 1.75; 2.65	Kanekasu	Surg. R2	− 1.81; 2.41	0.05; 1.52	0.744	0.820	0.734–0.880
		Anat. R1	2.81; 2.82		Anat. R2	3.16; 2.44	− 0.35; 1.46	*0.031*	0.846	0.771–0.898
	2D-CT	Surg. R1	− 0.46; 2.58	2D-CT	Surg. R2	− 1.32; 2.34	0.87; 1.21	*0.000*	0.878	0.817–0.920
		Anat. R1	3.06; 2.79		Anat. R2	2.99; 2.47	0.07; 1.17	0.605	0.901	0.851–0.935
Surg. vs. anat.	Kanekasu	Surg.	− 1.78; 2.41	Kanekasu	Anat.	2.98; 2.53	− 4.76; 1.07	*0.000*	0.907	0.859–0.939
	2D-CT	Surg.	− 0.89; 2.39	2D-CT	Anat.	3.02; 2.57	− 3.91; 0.82	*0.000*	0.946	0.917–0.965
Kanekasu vs. 2D-CT	Kanekasu	Surg.	− 1.78; 2.41	2D-CT	Surg.	− 0.89; 2.39	− 0.89; 1.76	*0.000*	0.731	0.611–0.818
		Anat.	2.98; 2.53		Anat.	3.02; 2.57	− 0.04; 1.77	0.839	0.760	0.651–0.838
With 3D-CT total	Kanekasu	Surg.	− 1.78; 2.41	3D-CT	Anat.	3.15; 2.93	− 4.92; 2.71	*0.000*	0.489	0.305–0.638
		Anat.	2.98; 2.53				− 0.16; 2.4	0.537	0.614	0.459–0.733
	2D-CT	Surg.	− 0.89; 2.39				− 4.04; 1.95	*0.000*	0.733	0.615–0.820
		Anat.	3.02; 2.57				− 0.13; 1.65	0.494	0.821	0.736–0.881

A systematic difference was found in Kanekasu view radiograph (− 4.76 ± 1.07°) and 2D-CT (− 3.91 ± 0.82°) between the measurements using the surgical and anatomical TEA (*p* < 0.001). The means of measurements using sTEA for determination of the posterior condyle angle significantly differ between Kanekasu view radiograph and 2D-CT as well as in comparison with the gold standard 3D-CT (*p* < 0.001).

Table 2 presents a comparison of the reliabilities between the different measurement methods. For R1, the intrarater reliability was significantly better for 2D-CT than Kanekasu view radiograph for measuring both the surgical (*p* < 0.01) and anatomical posterior condyle angles (*p* < 0.05). For R2, the intrarater reliability was significantly better for Kanekasu view radiograph than 2D-CT for measurement of the surgical posterior condyle angle (*p* < 0.05), however there was no significant difference between the ICCs for the anatomical posterior condyle angle. For R1, intrarater reliability was higher when using the aTEA than the sTEA in Kanekasu view radiograph (*p* < 0.05) and 2D-CT (*p* = 0.054, barely not significant (n.s.)), however for R2 there was no superiority found. Looking at the difference in intrarater reliability between the two raters, the more experienced rater (R2) had a higher intrarater reliability than R1 for the measurement of both anatomical and surgical angles on Kanekasu view radiograph (sTEA *p* < 0.001; aTEA *p* = 0.087). This pattern was not seen with 2D-CT however, where there was either no difference or the less experienced reviewer showed a higher intrarater reliability (*p* < 0.05).

**Table 2:** Comparison of reliabilities expressed as ICCs (intraclass correlation coefficient) between the three imaging modalities and anatomical versus surgical angles using Fisher z-transformation. A direction was given (>) for *p* values of *p* < 0.1. R1, 2; rater 1 and 2. Statistically significant p-values are shown in italics

Value 1	ICC	Value 2	ICC	*z*	Direction	*p*
Intrarater reliability (Kanekasu vs. 2D-CT)						
Kanekasu surg. R1	0.72	2D CT surg. R1	0.87	2.67	2D > Kanekasu	*0.004*
Kanekasu anat. R1	0.85	2D CT anat. R1	0.92	2.09	2D > Kanekasu	*0.018*
Kanekasu surg. R2	0.92	2D CT surg. R2	0.86	1.86	Kanekasu >2D	*0.031*
Kanekasu anat. R2	0.90	2D CT anat. R2	0.85	1.12	=	0.130
Intrarater reliability (surg. vs. anat.)						
Kanekasu surg. R1	0.72	Kanekasu anat. R1	0.85	2.19	anat. > surg.	*0.014*
2D CT surg. R1	0.87	2D CT anat. R1	0.92	1.34	anat. > surg.	0.054
Kanekasu surg. R2	0.92	Kanekasu anat. R2	0.90	0.73	=	0.231
2D CT surg. R2	0.86	2D CT anat. R2	0.85	0.23	=	0.408
Intrarater reliability (R1 vs. R2)						
Kanekasu surg. R1	0.72	Kanekasu surg. R2	0.92	4.28	R2 > R1	*< 0.001*
Kanekasu anat. R1	0.85	Kanekasu anat. R2	0.90	1.36	R2 > R1	0.087
2D CT surg. R1	0.87	2D CT surg. R2	0.86	0.25	=	0.401
2D CT anat. R1	0.92	2D CT anat. R2	0.85	2.09	R1 > R2	*0.018*
Interrater reliability (Kanekasu vs. 2D CT)						
Kanekasu surg.	0.82	2D CT surg.	0.88	1.38	2D > Kanekasu	0.084
Kanekasu anat.	0.85	2D CT anat.	0.90	1.12	=	0.130
Interrater reliability (surg. vs. anat.)						
Kanekasu surg.	0.82	Kanekasu anat.	0.85	0.62	=	0.266
2D CT surg.	0.88	2D CT anat.	0.90	0.61	=	0.272
Comparison with 3D (Kanekasu vs. 2D CT)						
Kanekasu surg.	0.49	2D CT surg.	0.73	2.47	2D > Kanekasu	*0.007*
Kanekasu anat.	0.61	2D CT anat.	0.82	2.81	2D > Kanekasu	*0.002*
Comparison with 3D (surg. vs. anat.)						
Kanekasu surg.	0.49	Kanekasu anat.	0.61	1.09	=	0.139
2D CT surg.	0.73	2D CT anat.	0.82	1.43	anat. > surg.	0.076

The interrater reliability was higher for 2D-CT than Kanekasu view radiograph for the surgical posterior condyle angle but without significance (*p* = 0.084). There was no superiority found for the interrater reliability using sTEA or aTEA in Kanekasu view radiographs and 2D-CT.

For both the surgical and anatomical posterior condyle angles (*p* < 0.01), the correlation with the gold standard 3D-CT is significantly higher in 2D-CT than Kanekasu view radiograph. Measurements based on 2D-CT using the aTEA correlated more closely with the 3D-CT measurement than using the sTEA, however *p* value was below significance (*p* = 0.076).

**Fig. 4:**
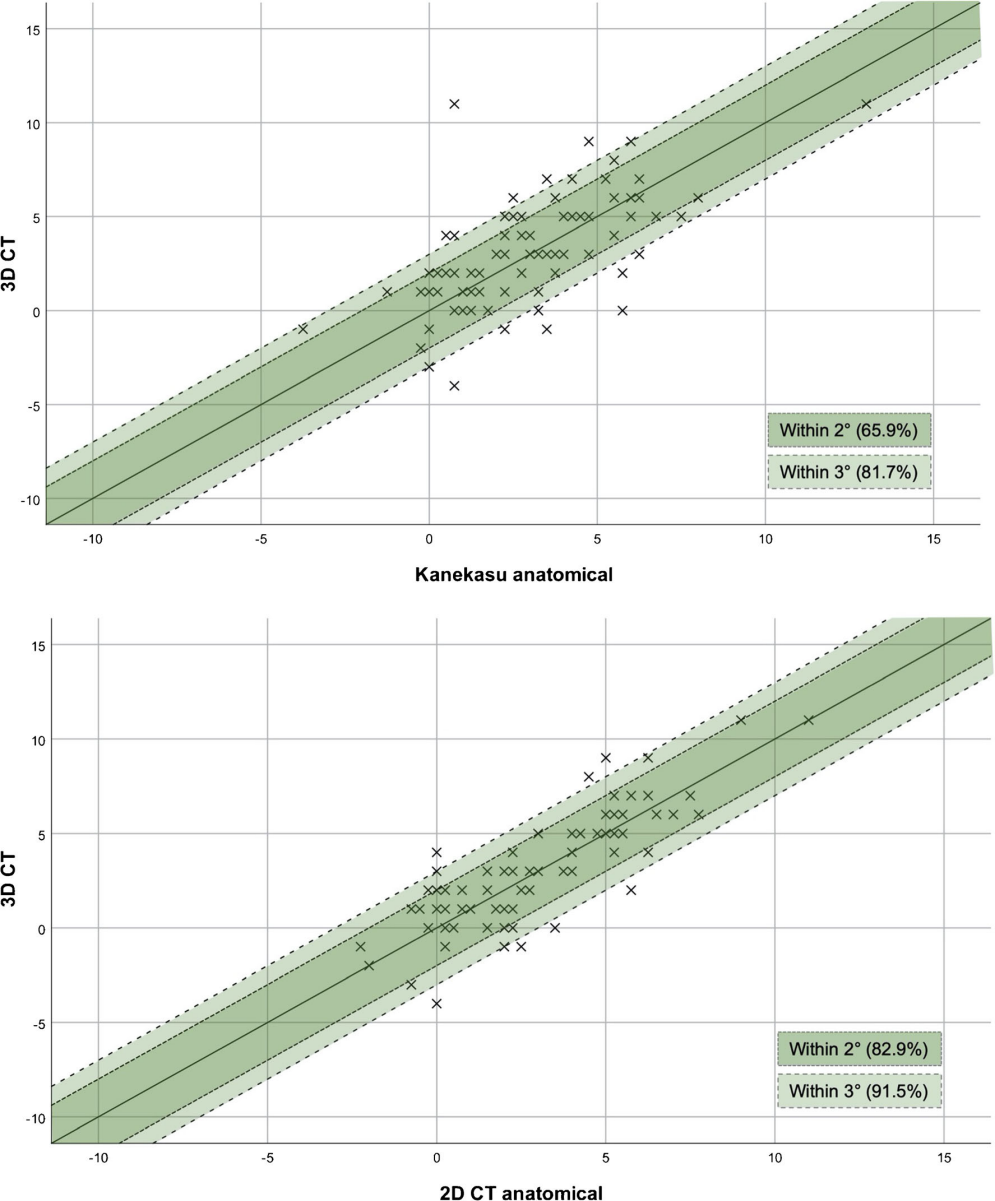
Graphs demonstrating the proportion of anatomical posterior condyle angle measurement values by Kanekasu view radiograph (top image) and 2D-CT (bottom image) within 2 or 3° of the gold standard 3D-CT measurement. The inner area shaded dark green represents values within 2°, and the outer light green area extends to 3°

Table 3 shows the probability of predicting the true value (3D-CT) using the aTEA in the imaging modalities investigated in this study within a certain variability of degrees. While the Kanekasu view radiograph predicts the true angle only in 65.9% of the cases, 2D-CT can measure the true angle with 82.9% certainty, provided that ± 2° variability from the true value is tolerated (Fig. 4). The percentages rise to 81.7% and 91.5% when ± 3° variability are accepted.

**Table 3:** Percentage of correct prediction of the true value (3D-CT = gold standard) using Kanekasu view radiographs and 2-dimensional computer tomography (2D-CT) within 2 or 3 degrees of variability. The percentages refer to measurements based on the anatomical transepicondylar axis

Predicting	within ± 2°	within ± 3°
2D-CT by Kanekasu	66/82 = 80.5%	75/82 = 91.5%
3D-CT by Kanekasu	54/82 = 65.9%	67/82 = 81.7%
3D-CT by 2D-CT	68/82 = 82.9%	75/82 = 91.5%

## Discussion

The most important findings and implications of this study were the following:

Firstly, the intra- and interrater reliability for measurements of the anatomical and surgical posterior condyle angle using Kanekasu view radiograph and 2D-CT were good to excellent according to ICC; highest ICCs were found for R1 when the aTEA was used, and for R2 when both aTEA and sTEA were used. This implies that measurements based on aTEA are easier to replicate compared to sTEA. This could be explained by the morphology of the anatomical landmarks used to determine the angles. For a less experienced rater, it might be more difficult to exactly identify the deepest point of the crescent-shaped sulcus of the medial epicondyle (sTEA) than the most prominent tip of the medial epicondyle (aTEA). This assumption can be confirmed by the intrarater reliability found in this study showing a superiority of the aTEA for referencing compared to sTEA for R1. The difficulty in identifying this landmark has been reported previously [19, 24], and difficulties may include flattening or bone formation over the median epicondyle [24]. A study evaluating CT images of osteoarthritic knees by Yoshino et al. [16] reported that the more severe the osteoarthritis, the more difficult it becomes to identify the medial sulcus, whereas the most prominent part of the medial epicondyle (used for the anatomical angle) remained detectable in all knees. Accordingly, Kanekasu et al. [19] did not use the sTEA for their measurements because the medial sulcus was difficult to identify.

Our results may also suggest that the experience of the rater is a decisive factor for the reliability of the measurements performed. The intrarater reliability analysis revealed that for inexperienced raters 2D-CT measurements are easier to replicate than measurements done in Kanekasu view radiographs. On the contrary, experienced raters are better at correctly replicating measurements based on Kanekasu view radiographs, especially when using the sTEA as a reference. Konigsberg et al. [25] made similar observations regarding the intrarater reliability in favour of less experienced residents when evaluating TKA component rotation using 2D-CT. An explanation for this could be that less experienced examiners may be more likely to reduce the metal artefacts in CT scans by optimising the contrast and brightness settings and thus achieve a higher resolution and visibility of the structures than experienced radiologists who may not take the time to do this. However, on the basis of the data collected in this study, it cannot be entirely ruled out that the variability between the readers could not also happen between readers of equal experience.

Interrater reliability analysis revealed a slightly higher reproducibility of 2D-CT measurements for the sTEA (n.s.). However, in direct comparison no significant difference could be found between the use of aTEA or sTEA for posterior condyle angle measurements in Kanekasu view radiographs and 2D-CTs (Table 2). These results suggest that neither the use of the surgical nor the anatomical TEA has a significant influence on the interrater reliability.

Our results are also in line with previous data of Kanekasu et al. [19] who analysed the interobserver differences in 50 knees between three observers. They showed interobserver variations of ± 0.7 to ± 1.0° [19]. In contrast, lower overall ICCs were reported by Konigsberg et al. [25]. They reported a good intrarater reliability (ICC = 0.61) and a poor interrater reliability (ICC = 0.39) for rotational measurements of the femoral component. As a consequence of the variable measurements, the authors questioned if commonly used axial CT images are the best diagnostic tool for the assessment of femoral component rotation.

The mean values of surgical and anatomical posterior condyle angles measured in this study correspond to previous findings [26]. Witoolkollachit et al. [26] analysed 40 knees after TKA using postoperative CT scans and measured both angles. The mean ± SD of the surgical and anatomical posterior condylar angle was − 1.34° ± 1.57° and 2.39° ± 2.80°, respectively. It is generally agreed that the “optimal” axial femoral component position should be between 0 and 5° external rotation to the sTEA [21, 27, 28]. In our study, we have measured surgical posterior condyle angles between − 0.46 ± 2.58 in 2D-CTs and − 1.81 ± 2.41 in Kanekasu view radiographs.

Secondly, a systematic difference was found between the surgical and the anatomical posterior condyle angle in both Kanekasu view radiograph (− 4.76 ± 1.07°) and 2D-CT (− 3.91 ± 0.82°). This significant difference was also found in comparison with 3D-CT measurements (− 4.92 ± 2.92 for Kanekasu; − 4.04 ± 1.95 for 2D-CT), which are based on the aTEA. These findings are in keeping with accepted principles [16, 26, 29, 30]. A recent study of Jang et al. [29] evaluated various reference axes of the femur based on 2128 CT scans. They found that the aTEA differed from the sTEA by a mean of 2.05° ± 1.33°. Other authors measured a mean difference between the surgical and anatomical TEA of 3.1° ± 1.0° and 3.98° ± 1.05° in CT scans of TKA patients [16, 26].

Thirdly, in comparison with the gold standard 3D-CT, 2D-CT showed a significantly higher correlation with 3D-CT than the Kanekasu view radiograph for both the sTEA and aTEA.

Measurements based on the aTEA correlated more strongly with the 3D-CT measurement than the sTEA. This is to be expected as the 3D measurement uses the anatomical transepicondylar axis for referencing [12]. In a landmark study in 2011, Hirschmann et al. [17] compared the intra- and interobserver reliability of conventional axial 2D and 3D-CT images; inter-observer reliability was significantly higher in 3D-CT compared to measurements in 2D-CT (ICC = 0.92 vs. ICC = 0.29, *p* < 0.001) and intraobserver reliability was significantly better in 3D-CT (ICC = 0.73 vs. ICC = 0.60, *p* < 0.001). They recommended the use of 3D-CT for assessing the femoral rotational component which is more reproducible than plain radiographs or 2D-CT [17]. Thus, the results of this study are in accordance with previous results from Hirschmann et al. [17]

Fourthly, the percentage of correct prediction of the true 3D-CT measurement was calculated and showed 65.9% and 82.9% with *a* ± 2° variability and 81.7% and 91.5% with *a* ± 3° variability certainty by the Kanekasu view radiograph and 2D-CT, respectively*.* A variability of 2° and 3° correspond to the accuracy of measurements using the gold standard 3D-CT; the median differences of femoral component position for intra- and interobserver testing is 2° and 3° with a range of 0–6° [31].

Several limitations of the present study have to be acknowledged. The study presents data of a relatively heterogeneous and small cohort of 82 knees from 27 male and 51 female patients the age of 24–83 years who underwent primary TKA between 2004 and 2019. Only two raters were used. Although the statistical power was adequate, larger studies with more patients or raters, and a more homogeneous sample, would help to determine how well our findings might generalise to others. A further limitation of this study is that for the 2D-CT measurements the axes were plotted on only one slice, whereas Kanekasu et al. [19] noted that the posterior margins of the medial and lateral condyles were not always in the same slice. The authors therefore measured the angle based on superimposed images of three consecutive slices traversing the epicondyles [19]. This method could potentially be more accurate than the single slice method used in this study. Another limitation is the restriction to pure radiological data assessment without considering clinical parameters. The clinical relevance of these data can only be derived from previous studies. Furthermore, it would be interesting to compare our data with measurements from asymptomatic patients after TKA. Future studies should correlate clinical parameters with various imaging modalities and assess PCA measurements from patients without symptoms.

The strength of this study is that the measurements are objective readings by two blinded raters using two established measurements techniques and protocols to assess femoral TKA component malrotation [5, 19]. ICCs of the two techniques were compared with the gold standard 3D-CT, and superiorities of the respective imaging modalities were stated depending on the experience of the rater.

## Conclusion

The intra- and interrater reliability for measurements of the anatomical posterior condyle angle for both the Kanekasu view radiograph and the 2D-CT were excellent for both raters. This implies that measurements using the aTEA are easier to replicate compared to the sTEA. In comparison with the gold standard 3D-CT, 2D-CT showed a significantly higher correlation with 3D-CT than the Kanekasu view radiograph measurements. The true 3D-CT value can be predicted with a certainty of 65.9% by the Kanekasu view radiograph and 82.9% by 2D-CT measurements with ± 2° variability. Hence, it can be concluded, that Kanekasu view radiograph is not as good as CT to determine the PCA. If 3D-CT is available, it should be preferred over 2D-CT and Kanekasu view radiograph for femoral component rotation measurements.
